# The Impact of Sleep on Sensory Processing in Typically Developing Children: Insights from Cross-Sectional and Longitudinal Data

**DOI:** 10.3390/children12020153

**Published:** 2025-01-28

**Authors:** Sophie Bellemare, Gabriela López-Arango, Florence Deguire, Inga S. Knoth, Sarah Lippé

**Affiliations:** 1 Department of Psychology, University of Montreal, Marie-Victorin Building, 90 Vincent-D’Indy Avenue, Montreal, QC H2V 2S9, Canada; 2 CHU Sainte-Justine Research Center, University of Montreal, 3175 Chemin de la Côte-Sainte-Catherine, Montreal, QC H3T 1C5, Canada

**Keywords:** children, sensory processing, sleep, child development

## Abstract

Background/Objectives: Previous research suggests that sleep quality and duration may significantly impact sensory experiences, yet the specific relationships in healthy early childhood remain unclear. This study explores the relationship between sleep parameters and sensory processing outcomes in typically developing children followed longitudinally from 3 to 12 months to 4 years of age. Methods: We assessed sleep problems, sleep duration, sleep onset latency, parasomnias, and sensory processing in a sample of typically developing children (N = 85). Sleep parameters were reported by parents, and sensory processing was evaluated using the Sensory Processing Measure—Parent version (SPM-P). Cross-sectional and longitudinal analyses examined predictors of sensory outcomes based on sleep patterns at 3–12 months, 18 months, 2 years, and 4 years of age. Results: Our findings indicate that greater sleep problems correlate with heightened sensory sensitivity across modalities, including touch and vision, as well as higher-order cognitive functions such as planning and social interactions. Specifically, sleep problems at 18 months were significant predictors of sensory processing at 4 years, while sleep duration at 2 years predicted planning skills. Additionally, longer sleep duration during infancy (3–12 months) positively influenced social participation at 4 years. Conclusions: This study underscores the critical role of sleep in shaping sensory processing outcomes in early childhood. Promoting healthy sleep habits may mitigate sensory processing difficulties, ultimately supporting emotional and social development.

## 1. Introduction

An increasing body of research highlights the critical role of sleep quality and quantity in child development, including emotional regulation, behavior, cognitive abilities, sensory–motor development, and brain maturation [1,2,3]. Moreover, sleep deprivation and poor sleep quality can lead to detrimental short- and long-term effects, including short-term memory, executive functioning, and some aspects of attention and lower performance in school-related tasks [3,4,5,6]. During early childhood—a period marked by rapid and significant developmental changes—adequate sleep, which occupies a significant portion of an infant’s day, plays a central role in cognitive, physical, and psychosocial well-being [7,8,9]. With sleep disturbances affecting approximately 25% of typically developing children and 50–80% of children with neurodevelopmental disorders [10,11], understanding how sleep influences developmental outcomes is crucial [8,12].

Given sleep’s essential role in child development, it is essential to establish clear expectations for optimal health and functioning. Sleep habits evolve quickly in early childhood, initially influenced by hunger rather than light cues [13,14], with circadian rhythms emerging between two and three months of age [15,16]. The recommended sleep duration varies by age, with newborns (0–3 months) needing 14–17 h, infants (4–11 months) requiring 12–15 h, toddlers (1–2 years) needing 11–14 h, and preschoolers (3–5 years) being recommended to get 10–13 h per night [17,18]. In terms of sleep quality, although there is no universally agreed-upon definition, evidence-based indicators from the National Sleep Foundation (2017) suggest that good sleep includes a sleep onset latency of less than 30 min, fewer than two nighttime awakenings lasting more than five minutes, and an efficiency defined as the ratio of total sleep time to time spent in bed of 85% or higher [19]. Additionally, parasomnias, defined as undesirable events or experiences during sleep [20], are fairly common in children but typically do not severely impact sleep quality or duration [21]. However, in severe cases, they can lead to further sleep issues [22]. Research has linked parasomnias to separation anxiety and increased inattention and hyperactivity [21], but studies on their developmental impact in childhood are limited.

While much research has focused on sleep’s impact on neurodevelopment, its connection to sensory processing remains less explored. Sensory processing refers to the brain’s ability to receive, organize, and respond to sensory input (visual, auditory, tactile, taste, smell, proprioception, and vestibular) [23,24]. Disruptions in these processes can lead to sensory processing disorders such as hyposensitivity, hypersensitivity, or sensory-seeking behaviors, which affect daily tasks, social participation, and independence [25,26]. Sensory processing difficulties are more prevalent in individuals with neurodevelopmental disorders, affecting 20–95% [27,28], but also occur in 3–16% of typically developing children [27,29,30].

Recent studies suggest that sensory processing can interact with sleep problems in clinical populations, including children with neurodevelopmental disorders such as ASD [31] and ADHD [32] as well as in those with fetal alcohol spectrum disorder [33] and preterm children [34]. Environmental changes during bedtime, such as noise or lighting, may overwhelm sensitive children, leading to bedtime resistance, prolonged sleep onset latency, and frequent awakenings. Thus, these challenges can significantly contribute to sleep problems [31,33,35]. Although only few researchers have addressed sleep and sensory processing in typically developing children [36,37,38,39,40,41], the emerging evidence suggests that children with sensory hypersensitivity traits tend to have more sleep problems than those with average sensory processing [40].

Given the existing research on sleep and sensory processing in clinical populations, it is essential to also investigate these relationships in neurotypical children. While previous studies have highlighted associations between sleep disturbances and sensory issues, most research has focused on cross-sectional data [36,37,38,39,40], which limits conclusions about causality. To date, only one longitudinal study has explored the relationship between sleep and sensory processing in typically developing young children [39]. However, the limited age range of 6 months to 2.5 years raises uncertainties regarding the classification of all participants as typically developing, as some may not have received formal diagnoses for potential neurodevelopmental disorders. Furthermore, some authors propose a potentially bidirectional relationship between sleep and sensory processing [42,43,44], highlighting the need for longitudinal studies to clarify these dynamics.

This study aims to investigate the relationship between sleep and sensory processing in typically developing children aged four years old using both cross-sectional and longitudinal data. We hypothesized that shorter sleep duration, indices of greater sleep problems, a longer sleep onset latency, and a higher frequency of parasomnias in infancy would be associated with poorer ratings (scale scores) of sensory processing at four years old and that these relationships would also be demonstrated cross-sectionally. Previous studies have suggested similar associations between sleep disturbances and sensory processing difficulties, particularly in clinical populations (e.g., ASD and ADHD) [31,32] and during early childhood [37,39]. Additionally, we sought to examine whether the effects of sleep disturbances during infancy manifest differently across sensory modalities, as different sensory systems may be uniquely susceptible to sleep-related disruptions. Furthermore, we aimed to explore whether the relationship between sleep parameters and sensory processing is influenced by covariables such as sex and socioeconomic status.

## 2. Materials and Methods

### 2.1. Participants and Procedures

We recruited 128 typically developing children (70 males) aged between 3 and 12 months to participate in this longitudinal study. Children were recruited at CHU Sainte-Justine’s birth unit and medical imaging department, in daycares and through social networks. Developmental and socioeconomic information was gathered from an in-house developmental questionnaire completed by the parents. All children were born at full term (>37 weeks) with no pregnancy or delivery complications. They had no significant health problems or suspicions of developmental delay. Parents gave informed written consent for themselves and for their infants prior to the study. The study was approved by the ethics, administrative, and scientific committees of the Ste-Justine’s University Hospital Research Center and all experiments were performed in accordance with relevant guidelines and regulations.

This project is a longitudinal study including three visits to CHU Sainte-Justine at the *Neuroscience of Early Development (NED)* laboratory. The first visit took place during the infant’s first year of life (between 3 and 12 months), during which the Ages and Stages Questionnaire, Third Edition (ASQ-3) [45] was administered to evaluate children’s developmental progress in five developmental areas (i.e., communication, gross motor, fine motor, problem solving, and personal–social). At 18 months, a follow-up was conducted with the parents through online questionnaires, including a second administration of the ASQ-3 [45]. The second visit occurred at two years of age, where the Bayley Scales of Infant and Toddler Development, Third Edition (Bayley-III) [46] was used to assess children’s cognitive, motor, and language development. At the third visit, when the children were four years old, an assessment of intellectual abilities was performed with the Wechsler Preschool and Primary Scale of Intelligence—Fourth Edition (WPPSI-IV) [47], allowing us to obtain a Full-Scale IQ (FSIQ). A subsample of 85 typically developing children, for whom the variables of interest were available, was included in the analyses (see Table 1 for demographics).

### 2.2. Measure

#### 2.2.1. Sleep Duration and Sleep Onset Latency

The parents of each participant completed an in-house questionnaire covering the child’s overnight sleep patterns at each visit (between 3 and 12 months, two years, and four years of age) and at the follow-up (18 months of age). Bedtime and wake time were provided by the parent, and nighttime sleep duration was calculated based on these times. Additionally, the parent also reported the child’s sleep onset latency, which is the time it takes for the child to fall asleep.

#### 2.2.2. Sleep Problems and Parasomnias

At 18 months, 2, and 4 years of age, the parents completed the Child Behavior Checklist 1.5–5 years old (CBCL 1.5–5 years old), a reliable and valid measure for behavioral problems [48]. Parents rate each behavior on a three-point Likert scale (0—not true, 1—somewhat or sometimes true, or 2—very or often true). Sleep problems were assessed using five items measuring dyssomnia symptoms (doesn’t want to sleep alone; has trouble getting to sleep; resists going to bed at night; sleeps less than most kids during day and/or night; wakes up often at night), which has been utilized in previous research to evaluate sleep-related issues in children [49,50,51]. A total sleep score, ranging from 0 to 10, was calculated by summing the scores across all items, with higher scores indicating greater sleep problems. In addition to this, two parasomnia items (has nightmares; talks or screams in sleep) were used to create a separate parasomnia scale. The Sleep Problems Scale demonstrates test–retest reliability (r = 0.92 over 8 days) and internal consistency (α = 0.78), according to the psychometric data presented in the CBCL manual [52].

While the CBCL sleep scale is not specifically validated as a standalone sleep measure, it has been shown to correlate with other objective and subjective sleep measures, including sleep diaries, actigraphy, polysomnography, and validated questionnaires such as The Children’s Sleep Habits Questionnaire [53,54]. These studies demonstrated that the CBCL is a reliable tool for assessing global sleep functioning in children, making it an appropriate choice for studies aiming to explore sleep-related concerns in pediatric populations.

#### 2.2.3. Sensory Processing

At the third visit, the parents completed the Sensory Processing Measure–Preschool questionnaire (SPM-P) home form [55]. The SPM-P is a 75-item standardized rating scale designed to assess sensory processing difficulties, praxis, and social participation in children aged 2 to 5 years [55]. The SPM-P items are divided across several sensory domains, each reflecting potential problems with sensory responsiveness: difficulties with responsiveness to visual stimuli (vision), auditory stimuli (hearing), tactile stimuli (touch), proprioception (body awareness), and vestibular processing (balance and motion). In addition to these sensory domains, the SPM-P also assesses higher-level functional domains such as praxis (planning and ideas) and social participation. The tool provides a total sensory systems score, summarizing performance across the sensory domains. The SPM-P is completed by the child’s parent or caregiver and takes approximately 15–20 min to complete. Parents report their child’s typical behavior over the past month using a 4-point Likert-type response scale ranging from never (1) to always (4). A higher raw score on the SPM-P is suggestive of greater dysfunction across all subscales. To describe the sample (see Table 2), T scores were used [55]. For statistical analyses, raw scores were used to maximize variability, as the sample was not expected to show sensory processing issues. The SPM-P home form demonstrates test–retest reliability (r = 0.90–0.94) and internal consistency (α = 0.75–0.93), according to its psychometric validation [56].

### 2.3. Statistical Analysis

Data were analyzed using IBM SPSS Statistics (Version 29; IBM Corp., Armonk, NY, USA). First, the data were explored to ensure assumptions of normality were met. The initial analysis also involved descriptive statistics for demographic and sleep data. Then, to determine the inter-relatedness between sleep at four years old (sleep duration, sleep onset latency, sleep problems (CBCL), and parasomnias (CBCL)) and sensory processing (subscales from the SPM-P), bivariate Pearson correlations were computed prior to the regression models. The correlation coefficients yielded the maximum degree of linear relationship that could be obtained between our variables. Correlations were conducted with neurodevelopmental measures at each assessment point to determine if these variables could impact the results. Specifically, neurodevelopment was assessed using the ASQ-3 during the first time point and at 18 months, the Bayley-III at 2 years, and the WPPSI-IV at 4 years. If a significant correlation was revealed in these preliminary analyses, the corresponding neurodevelopmental variable was added as an independent variable in the main regression models. Finally, multiple linear regression analyses were performed to examine how sleep at four years of age could predict sensory processing at four years old. Seven regression models were built, each focusing on different SPM-P subscales. The models included covariates such as sex and socioeconomic status (i.e., maternal education and income), while the sleep variables used in each model (sleep duration, sleep onset latency and sleep problems from the CBCL, and parasomnias from the CBCL) were selected based on their significance in the prior Pearson correlation analyses. Given the inter-related nature of the SPM-P subscales, a Bonferroni correction for multiple comparisons was applied.

In addition to the cross-sectional analyses, longitudinal analyses were performed to examine how sleep at earlier time points (3–12 months, 18 months, and 2 years) predicted sensory processing at 4 years old. Six regression models were built for these analyses, each including the same covariates as the cross-sectional models. When variables from the first visit (3–12 months) were included, the child’s age at the first visit was added as a covariate due to the variability in age at that time point. Bivariate Pearson correlations, multiple regression models, and Bonferroni correction were used to explore these relationships, following the same approach as for the cross-sectional analyses.

## 3. Results

### 3.1. Participant Characteristics and Descriptive Data

Of the total subsample (N = 85), 46 participants (54.1%) were male and 39 (45.9%) were female. Demographic details, along with child developmental assessments at different time points, are provided in Table 1, demonstrating that our sample consists of typically developing children. The mean sleep duration (with SDs in parentheses) in children was 11.0 (0.9) hours at age 3–12 months (range = 8.5–13.5 h), 11.2 (0.7) at 18 months (range = 9.5–13.0 h), 10.9 (0.7) at 2 years (range = 9.3–12.5 h), and 10.7 (0.6) at 4 years (range = 8.5–12 h). Based on the recommended sleep durations for each age group, our sample’s sleep duration appears to be on the lower end, with many children sleeping less than the suggested amount for optimal development [17,18]. The sleep onset latency (with SDs in parentheses) was 15 min (12) at age 3–12 months, 15 (12) at 18 months, 20 (14) at 2 years, and 30 (23) at 4 years. Sleep problems scores (with SDs in parentheses) ranging from 0 to 10 (higher scores indicating greater sleep problems) were 1.3 (1.6) at age 18 months, 1.8 (2.0) at 2 years, and 2.3 (2.3) at age 4 years. Parasomnia scores (with SDs in parentheses), ranging from 0 to 4 (higher scores indicating more frequent parasomnias), were 0.4 (0.7) at 18 months, 0.5 (0.8) at 2 years, and 0.7 (0.9) at 4 years. Sensory processing scores at age 4 years, derived from the SPM-P, are presented in Table 2.

### 3.2. Cross-Sectional Relationship

#### 3.2.1. Correlations Between Sleep and SPM-P Subscales

Bivariate Pearson correlation analyses were performed between the variables of interest at four years old (see Table 3). We found significant correlations between several sleep variables and the SPM–P subscale, including (1) SPM-P vision score with sleep onset latency (r = 0.25, *p* < 0.05) and CBCL sleep problems (r = 0.29, *p* < 0.01), (2) SPM-*p* touch score with CBCL sleep problems (r = 0.39, *p* < 0.001) and CBCL parasomnias (r = 0.26, *p* < 0.05), (3) SPM-P body awareness score with sleep onset latency (r = −0.23, *p* < 0.05), (4) SPM-P balance and motion score with CBCL sleep problems (r = 0.29, *p* < 0.01), (5) SPM-P total sensory systems score with CBCL sleep problems (r = 0.39, *p* < 0.001), (6) SPM-P planning and ideas score with sleep onset latency (r = 0.25, *p* < 0.05) and CBCL sleep problems (r = 0.40, *p* < 0.001), and (7) SPM-P social participation score with CBCL sleep problems (r = 0.26, *p* < 0.05).

#### 3.2.2. Regression Analysis Findings

**Figure 1:**
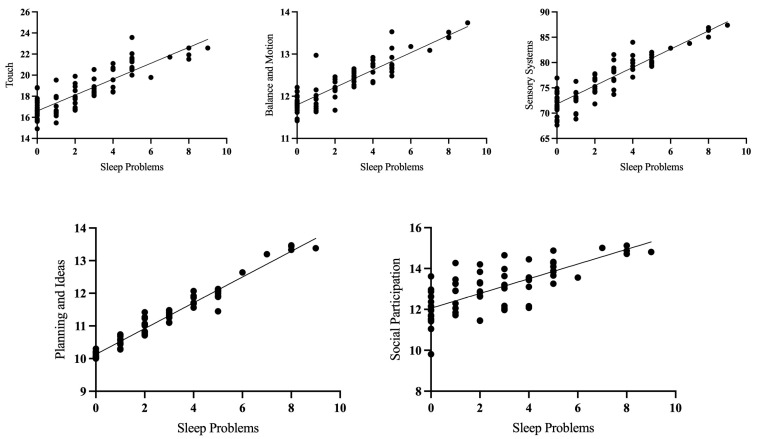
Scatterplots showing the relationship between sleep problems at visit 3 (4 years) and various sensory processing measures at visit 3 (4 years), controlling for covariates (sex, maternal education, and family income).

Multiple linear regression analyses were conducted to examine how sleep variables predicted different SPM–P subscales at four years old. Each regression model included sex, maternal education, and family income as covariates. CBCL sleep problems significantly predicted SPM-P touch (β = 0.35, *p* < 0.01), balance and motion (β = 0.32, *p* < 0.01), total sensory systems (β = 0.39, *p* < 0.001), planning and ideas (β = 0.36, *p* < 0.01) and social participation (β = 0.25, *p* < 0.05) scores. Across all subscales, greater sleep problems predicted greater dysfunction in sensory processing, as well as poorer planning and reduced social participation (See Figure 1). Sleep onset latency and CBCL parasomnias did not significantly predict any of the SPM-P subscales. Maternal education was a significant predictor of SPM-P vision score (β = −0.39, *p* < 0.01), with lower maternal education predicting greater dysfunction. See Table 4 for the detailed results of the linear regression analyses.

### 3.3. Longitudinal Relationship

#### 3.3.1. Correlations Between Sleep and SPM-P Subscales

Bivariate Pearson correlation analyses were performed between sleep variables at 3–12 months, 18 months, and 2 years, and sensory processing variables at 4 years old (see Table 5). We found significant correlations between several sleep variables and the SPM–P subscale, including (1) SPM-P vision score with sleep duration at 2 years (r = −0.26, *p* < 0.05) and CBCL sleep quality at 18 months (r = 0.41, *p* < 0.001), (2) SPM-P touch score with CBCL sleep quality at 18 months (r = 0.37, *p* < 0.001) and CBCL parasomnias at 2 years (r = 0.24, *p* < 0.05), (3) SPM-P balance and motion score with CBCL sleep parasomnias at 2 years (r = 0.23, *p* < 0.05), (4) SPM-P total sensory systems score with CBCL sleep quality at 18 months (r = 0.37, *p* < 0.001) and CBCL sleep parasomnias at 2 years (r = 0.23, *p* < 0.05), (5) SPM-P planning and ideas score with sleep duration at 2 years (r = −0.40, *p* < 0.001), CBCL sleep quality at 18 months (r = 0.36, *p* < 0.001) and CBCL parasomnias at 2 years (r = 0.26, *p* < 0.05), and (6) SPM-P social participation score with sleep duration at 3–12 months (r = −0.22, *p* < 0.05).

#### 3.3.2. Regression Analysis Findings

**Figure 2:**
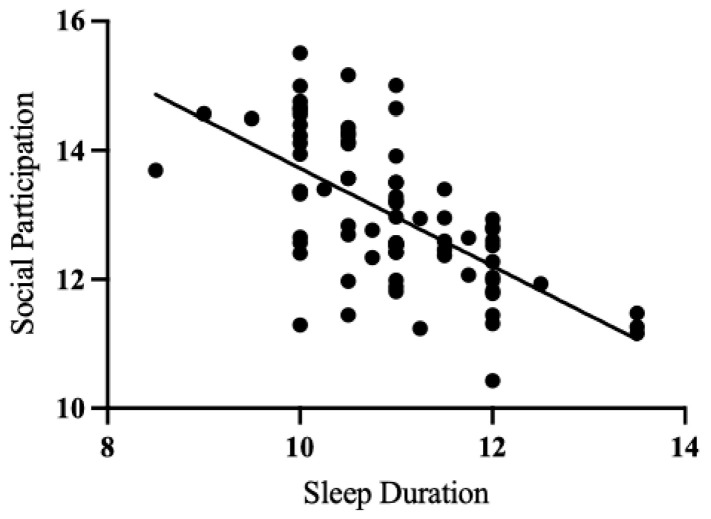
Scatterplot illustrating the relationship between nighttime sleep duration at visit 1 (3–12 months) and predicted social participation scores at visit 3 (4 years), controlling for covariates (sex, maternal education, family income, and age at visit 1).

Multiple linear regression analyses were conducted to examine how sleep variables at 3–12 months, 18 months, and 2 years predicted different SPM–P subscales at 4 years old. Each regression model included sex, maternal education, and family income as covariates. Sleep duration at 3–12 months significantly predicted SPM-P social participation (β = −0.25, *p* < 0.05) score, indicating that longer sleep was associated with greater social participation (see Figure 2).

**Figure 3:**
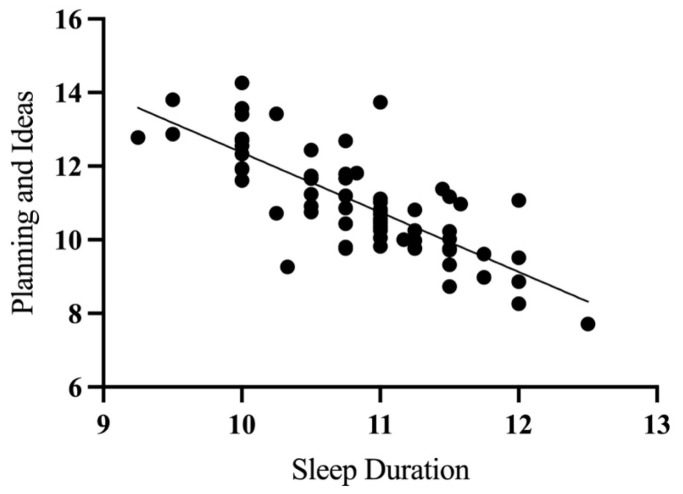
Scatterplot illustrating the relationship between nighttime sleep duration at visit 2 (2 years) and predicted planning and ideas scores at visit 3 (4 years), controlling for covariates (sex, maternal education, family income, and Bayley-III—cognition).

Additionally, sleep duration at two years significantly predicted the SPM-P planning and ideas (β = −0.34, *p* < 0.01) score, with longer sleep linked to fewer difficulties in planning and ideas (see Figure 3).

**Figure 4:**
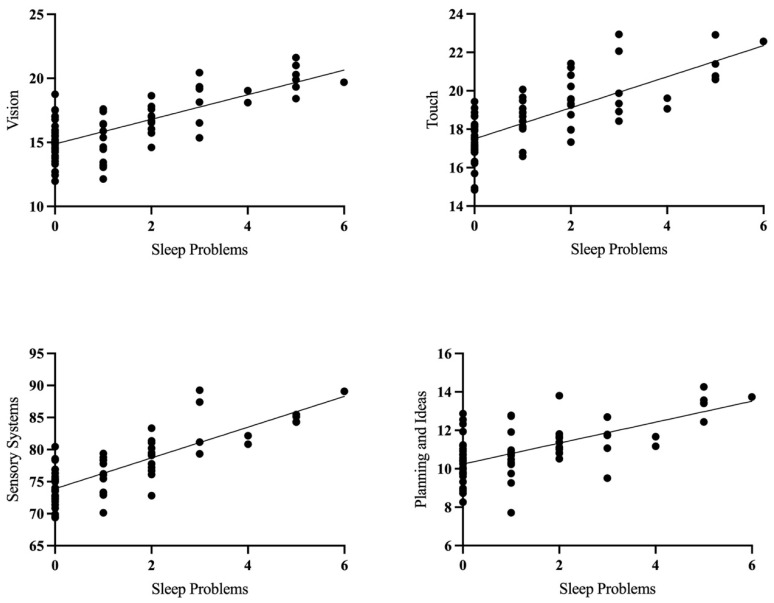
Scatterplots showing the relationship between sleep problems at follow-up (18 months) and various sensory processing measures at visit 3 (4 years), controlling for covariates (sex, maternal education, family income, and ASQ—problem resolution).

CBCL sleep problems at 18 months significantly predicted SPM-P vision (β = 0.41, *p* < 0.001), touch (β = 0.31, *p* < 0.01), total sensory systems (β = 0.34, *p* < 0.01), and planning and ideas (β = 0.28, *p* < 0.05) scores. Across all subscales, greater sleep problems predicted greater dysfunction in sensory processing, as well as poorer planning (see Figure 4).

CBCL parasomnias did not significantly predict any of the SPM-P subscales. Maternal education was a significant predictor of SPM-P vision score (β = −0.40, *p* < 0.001), with lower maternal education predicting greater dysfunction. See Table 6 for the detailed results of the linear regression analyses.

## 4. Discussion

This study aimed to investigate the relationship between sleep parameters and sensory processing outcomes in typically developing children aged four years, utilizing both cross-sectional and longitudinal data. The findings contribute valuable insights into the complex interplay between sleep and sensory processing, highlighting how early sleep patterns can significantly influence sensory experiences during childhood development. The results demonstrated that greater sleep problems were associated with concomitant heightened sensory sensitivity across multiple modalities, including touch, vision, and movement as well as higher-order cognition (planning and social). Children experiencing difficulties with sleep quality, such as frequent night awakenings or resisting going to bed, were more likely to exhibit hypersensitivity in sensory domains and struggle more with planning and social interactions. These findings are consistent with previous research that have identified similar patterns in both typically developing children and children with neurodevelopmental disorders [35,37,39,41].

Sleep problems at 18 months were also shown to be a predictor of sensory processing and planning and ideas at 4 years of age, and sleep duration at 2 years of age could predict planning and ideas measured at 4 years of age. These findings align with previous research that suggests sufficient sleep supports cognitive, motor, and emotional development [5]. The link between sleep duration and the outcome measure underscores the importance of adequate sleep during infancy as a foundational element for later developmental milestones. Moreover, our study revealed that longer sleep duration during infancy (3–12 months) was a significant predictor of social participation at 4 years of age. Studies report that a large proportion of sleep disturbances found in children presenting with a neurodevelopmental disorder at later age were also present very early on [57,58]. Our results highlight our capacity to identify children with suboptimal neurodevelopment in the social realm early on, and also in the general population.

Interestingly, sleep duration seemed to have more influence on higher-order sensory functions, such as social participation and planning, rather than lower-order sensory modalities like vision or hearing. This may be due to the integrative nature of social and planning functions, which rely on the successful coordination of multiple sensory systems [59]. Insufficient sleep may disrupt the ability to synchronize these complex sensory processes [60,61], leading to challenges in social interaction and motor planning, as shown in our findings. These results have practical implications for interventions aimed at improving sensory integration in children with sleep difficulties. By promoting better sleep habits from an early age, it may be possible to mitigate sensory processing difficulties, particularly in areas requiring higher cognitive and planning integration.

Based on Dunn’s sensory processing model, young children exhibiting sensory sensitivity have a low neurological threshold, meaning they tend to react more quickly and intensely to sensory input compared to their peers [62]. Sensory sensitivity, particularly hypersensitivity, can lead to challenges in daily functioning, as children may become overwhelmed by ordinary sensory input [62,63,64]. In our study, we found that sleep problems were linked to increased sensory sensitivity, which aligns with findings from other studies on typically developing children [37,39,40,41]. The connection between poor sleep and increased sensitivity suggests that the brain’s ability to filter and integrate sensory information may be compromised when sleep is inadequate or of poor quality. Moreover, research in the context of pain sensitivity has shown that sleep deprivation can heighten sensitivity to pain [65]. Supporting this connection, electroencephalogram studies utilizing evoked-response potentials indicate that individuals who experience poor sleep face more difficulties with sensory gating—the process of filtering out unnecessary sensory information—during the pre-sleep wakefulness period [42].

Our study also provided insights into specific sensory modalities that are particularly vulnerable to sleep disturbances. Children with greater sleep problems were more sensitive to visual and touch stimuli, suggesting that these types of processing might be especially susceptible to the effects of inadequate sleep. This finding invites further research into how sleep disturbances differentially impact various sensory systems, but it is interesting to mention that [39] found the same associations with vision and touch.

Building on these findings, it is essential to consider how inadequate sleep during early childhood can disrupt the maturation of sensory processing. Sleep is known to play a key role in neural plasticity and emotional regulation [7,61,66], both of which are critical for sensory processing and cognition [23]. During early childhood, sleep supports the maturation of neural networks that are responsible for integrating sensory inputs, allowing the child to respond appropriately to their environment [67,68]. Inadequate sleep, particularly during critical developmental windows, can hinder the proper development of these sensory systems. Early childhood is a period marked by significant neurodevelopment, where the brain’s ability to adapt and reorganize through plasticity is at its peak [69]. Sleep facilitates synaptic pruning, the process by which unnecessary neural connections are eliminated, while strengthening those that are most crucial for functioning [67,70,71]. If sleep is insufficient or of poor quality, this process can be disrupted, leading to less efficient neural networks and, by extension, poorer sensory integration and processing.

Another finding of this study was the moderating effect of a socioeconomic factor, maternal education, on the relationship between sleep and sensory processing. Children from more educated mothers had fewer sensory difficulties. A possible explanation is that they generally exhibited better sleep quality [72,73], which in turn predicted more favorable sensory processing outcomes. This suggests that children in more resource-rich environments may benefit from better sleep hygiene practices, leading to more regulated sensory processing.

Despite the strengths of this study, there are limitations that must be acknowledged. The reliance on parental reports for both sleep and sensory processing data may introduce bias, as parental perceptions can be subjective and influenced by personal expectations or stress. Future studies should aim to include objective measures of sleep, such as actigraphy, as well as standardized assessments of sensory processing to validate and enrich the findings. While longitudinal analyses provide a clearer picture of how early sleep patterns influence later sensory outcomes, it is important to note that our study only included a single time point for measuring sensory processing. As a result, we were unable to examine the potential reverse causality—how sensory processing may also impact sleep patterns over time. Future research should focus on further longitudinal work to understand how the relationship between sleep and sensory processing evolves throughout childhood and into adolescence. An additional avenue for exploration is the neurobiological mechanisms linking sleep to sensory processing. Emerging advances in neuroimaging techniques, such as functional MRI and EEG, could deepen our understanding of how sleep impacts brain structures and networks that support sensory integration in typically developing children. Combined with large, longitudinal datasets, such techniques can help uncover specific brain mechanisms involved in sleep problems and sensory processing, offering insights that are essential for designing interventions targeting both sleep and sensory processing challenges.

## 5. Conclusions

This study highlights the critical role of sleep in shaping sensory processing outcomes in early healthy childhood. By identifying sleep parameters that influence sensory sensitivity and the moderating effects of socioeconomic factors, it provides a framework for future studies and interventions. Promoting healthy sleep habits from an early age may support better sensory processing and contribute to cognitive, emotional, and social development. Policymakers, pediatricians, and educators should consider incorporating sleep education and interventions into early childhood programs. Further research into the intricate relationship between sleep and sensory processing is warranted to fully understand the long-term impacts of early sleep patterns on child development.

## Figures and Tables

**Table 1:** Characteristics of participants.

Measure	N	Mean (SD)	Range	% At Risk (ASQ-3)
Age				
Visit 1	85	5.94 (2.23)	3–12	
Follow-up	81	18.04 (1.04)	17–24	
Visit 2	84	24.26 (1.07)	23–29	
Visit 3	85	48.52 (1.31)	46–54	
Socioeconomic status				
Maternal education (years)	84	16.94 (2.94)	9–23	
Family income (USD)	84	137,815.48 (87,884.81)	20,000–700,000	
Head circumference (percentile)	85	79.54 (23.28)	15–99	
ASQ-3 (4 months)				
Communication	18	49.72 (6.52)	35–60	0.00
Gross Motor	18	52.22 (8.61)	35–60	5.56
Fine Motor	18	45.00 (13.17)	15–60	11.11
Problem Solving	18	51.39 (7.82)	35–60	0.00
Personal–Social	18	48.89 (10.37)	30–60	16.67
ASQ-3 (6 months)				
Communication	26	44.81 (11.09)	20–60	11.54
Gross Motor	26	40.19 (12.61)	15–60	7.69
Fine Motor	26	47.88 (13.13)	15–60	7.69
Problem Solving	26	50.96 (8.13)	35–60	0.00
Personal–Social	26	48.08 (13.57)	10–60	11.54
ASQ-3 (8 months)				
Communication	13	48.46 (9.66)	30–60	7.69
Gross Motor	13	43.85 (17.46)	15–60	30.77
Fine Motor	13	52.69 (9.71)	35–60	15.38
Problem Solving	13	52.69 (11.11)	20–60	7.69
Personal–Social	13	44.23 (17.30)	15–60	38.46
ASQ-3 (10 months)				
Communication	13	36.15 (12.10)	20–60	15.38
Gross Motor	13	31.15 (19.81)	5–60	53.85
Fine Motor	13	46.15 (14.60)	20–60	30.77
Problem Solving	13	44.23 (17.42)	10–60	15.38
Personal–Social	13	41.54 (13.45)	20–60	23.08
ASQ-3 (18 months)				
Communication	81	40.00 (15.00)	5–60	3.70
Gross Motor	81	54.57 (7.87)	20–60	2.47
Fine Motor	81	52.84 (8.36)	30–60	1.23
Problem Solving	81	43.33 (10.81)	15–60	11.11
Personal-Social	81	53.77 (11.61)	25–70	2.47
Bayley-III				
Cognitive	84	117.92 (16.04)	90–145	
Language	84	103.30 (11.77)	77–138	
Motor	84	105.65 (10.09)	88–145	
WPPSI-IV				
Full-Scale IQ	74	109.49 (12.14)	78–134	

**Table 2:** Sensory processing in 4-year-old children.

Variable	N	Mean (SD)	% Typical	% Some Problems	% Definite Dysfunction
**Sensory Systems**					
Vision	85	55.07 (8.38)	67.06	27.06	5.88
Hearing	85	54.74 (8.87)	70.59	22.35	7.06
Touch	85	51.91 (8.80)	78.82	18.82	2.35
Body awareness	85	54.49 (6.97)	69.41	30.59	0.00
Balance and motion	85	49.80 (7.16)	91.76	7.06	1.18
Total	85	54.19 (6.46)	78.82	20.00	1.18
**Higher-level sensory processing**					
Planning and ideas	85	49.94 (8.57)	83.53	16.47	0.00
Social participation	85	51.56 (8.03)	87.06	12.94	0.00

SPM-P scores are reported as T scores, standardized with a mean of 50 and a standard deviation of 10. Higher T scores reflect greater levels of dysfunction. According to the SPM-P classification, scores below 60 are considered “typical”, scores between 60 and 69 indicate “some problems”, and scores above 70 signify “definite dysfunction”.

**Table 3:** Pearson correlation coefficients between sleep variables and SPM-P subscales in 4-year-old children.

	SPM-P Subscales							
Sleep Variables	Sensory Systems					Higher-Level Sensory Processing		
	Vision	Hearing	Touch	Body Awareness	Balance and Motion	Total	Planning and Ideas	Social Participation
Sleep duration (h)	0.14	0.11	−0.04	0.09	0.04	0.09	−0.10	−0.17
Sleep onset latency (min)	0.25 *	0.13	0.22	−0.23 *	0.22	0.20	0.25 *	0.17
Sleep problems	0.29 **	0.19	0.39 ***	0.07	0.29 **	0.39 ***	0.40 ***	0.26 *
Parasomnias	0.07	0.06	0.26 *	0.08	0.03	0.17	0.07	0.00

* *p* < 0.05, ** *p* < 0.01, *** *p* < 0.001.

**Table 4:** Results of multiple linear regression analyses between sleep variables and SPM-P subscales in 4-year-old children.

	SPM-P Subscales						
Covariables and Sleep Variables	Sensory Systems				Higher-Level Sensory Processing		
	Vision	Touch	Body Awareness	Balance and Motion	Total	Planning and Ideas	Social Participation
Sex	0.04	−0.04	−0.16	0.11	−0.02	−0.02	−0.10
Maternal education (years)	−0.39 **	−0.12	−0.04	−0.11	−0.24 *	−0.07	0.20
Family income	−0.01	0.15	0.08	0.15	0.10	−0.03	0.04
Sleep onset latency (min)	0.16	n/a	−0.24 *	n/a	n/a	0.07	n/a
Sleep problems	0.20	0.35 **	n/a	0.32 **	0.39 ***	0.36 **	0.25 *
Parasomnias	n/a	0.19	n/a	n/a	n/a	n/a	n/a
R^2^	0.25 ***	0.21 **	0.09	0.11	0.20 **	0.17 *	0.12 *

* *p* < 0.05, ** *p* < 0.01, *** *p* < 0.001. Values in **bold** remained significant after Bonferroni correction.

**Table 5:** Pearson correlation coefficients between sleep variables from infancy and SPM-P subscales in 4-year-old children.

		SPM-P Subscales							
Sleep Variables		Sensory Systems					Higher-Level Sensory Processing		
		Vision	Hearing	Touch	Body Awareness	Balance and Motion	Total	Planning and Ideas	Social Participation
Sleep duration (h)	3–12 months	−0.01	−0.03	0.01	0.10	0.03	0.02	0.02	−0.22 *
	18 months	−0.22	−0.20	−0.16	0.10	−0.10	−0.20	−0.03	−0.17
	2 years	−0.26 *	−0.05	−0.13	0.08	−0.07	−0.15	−0.40 ***	−0.13
Sleep onset latency (min)	3–12 months	0.16	−0.06	−0.02	0.03	−0.06	0.04	0.07	−0.02
	18 months	0.09	0.03	0.05	−0.03	0.01	0.06	0.07	−0.09
	2 years	0.07	0.07	0.12	−0.06	0.08	0.11	0.04	0.05
Sleep problems	18 months	0.41 ***	0.12	0.37 ***	0.00	0.10	0.37 ***	0.36 ***	0.10
	2 years	0.18	0.03	0.17	−0.16	0.06	0.13	0.08	0.06
Parasomnias	18 months	0.12	0.06	0.20	0.00	0.14	0.16	0.06	0.04
	2 years	0.14	0.10	0.24 *	0.12	0.23 *	0.23 *	0.26 *	0.15

* *p* < 0.05, *** *p* < 0.001.

**Table 6:** Results of multiple linear regression analyses between sleep variables from infancy and SPM-P subscales in 4-year-old children.

		SPM-P Subscales					
Covariables and Sleep Variables		Sensory Systems				Higher-Level Sensory Processing	
		Vision	Touch	Balance and Motion	Total	Planning and Ideas	Social Participation
Sex		0.06	−0.03	0.08	0.00	−0.02	−0.17
Maternal education (years)		−0.40 ***	−0.06	−0.08	−0.21	−0.05	0.23
Family income		−0.02	0.12	0.14	0.09	−0.05	0.02
Age at visit 1		n/a	n/a	n/a	n/a	n/a	−0.05
ASQ—problem resolution	18 months	0.20	−0.05	n/a	0.08	0.10	n/a
Bayley—cognition	2 years	0.14	n/a	n/a	n/a	0.08	n/a
Sleep duration (h)	3–12 months	n/a	n/a	n/a	n/a	n/a	−0.25 *
	2 years	−0.20 *	n/a	n/a	n/a	−0.34 **	n/a
Sleep problems	18 months	0.41 ***	0.31 **	n/a	0.34 **	0.28 *	n/a
Parasomnias	2 years	n/a	0.16	0.25 *	0.14	0.17	n/a
R^2^		0.39 ***	0.17 *	0.07	0.20 *	0.29 **	0.13

* *p* < 0.05, ** *p* < 0.01, *** *p* < 0.001. Values in **bold** remained significant after Bonferroni correction.

## Data Availability

Anonymized individual-level data from the study including data dictionaries and data collection tools will be made available upon request. Requests for access will be reviewed by a data-access committee. The data are not publicly available due to the absence of authorization in consent forms allowing for the public publication of participants’ data.
